# PPARD +294C overrepresentation in general and long-lived population in China Bama longevity area and unique relationships between PPARD +294T/C polymorphism and serum lipid profiles

**DOI:** 10.1186/s12944-015-0016-3

**Published:** 2015-03-07

**Authors:** Chen-Yuan Luo, Cheng-Wu Liu, Lin Ge, Guo-Fang Pang, Ming Yang, Cai-You Hu, Ze-Ping Lv, Ning-Yuan Chen, Hai-Yan Li, Hua-Yu Wu, Yi-Yuan Wang, Rui-Xing Yin, Shang-Ling Pan, Jun-Hua Peng

**Affiliations:** Department of Pathophysiology, School of Preclinical Medicine, Guangxi Medical University, 22 Shuangyong Road, Nanning, Guangxi 530021 People’s Republic of China; Department of Neurology, Jiangbin Hospital of Guangxi Zhuang Autonomous Region, 85 Hedi Road, Nanning, Guangxi 530021 People’s Republic of China; Department of Cell Biology & Genetics, School of Preclinical Medicine, Guangxi Medical University, 22 Shuangyong Road, Nanning, Guangxi 530021 People’s Republic of China; Department of Cardiology, Institute of Cardiovascular Diseases, the First Affiliated Hospital, Guangxi Medical University, 22 Shuangyong Road, Nanning, Guangxi 530021 People’s Republic of China

**Keywords:** Peroxisome proliferator-activated receptor delta (PPARD), Longevity, Lipoprotein, Polymorphism, Association study

## Abstract

**Background:**

The +294T/C polymorphism in the peroxisome proliferator-activated receptor delta (PPARD) gene is associated with hyperlipidemia in several younger populations, but results are still inconsistence across ethnic groups and its possible impact on the lipid profiles of long-lived individuals remains unexploited. Here, we aimed to evaluate the possible correlation between PPARD +294T/C and serum lipid levels in a long-lived population in Bama, a region known for longevity situated in Guangxi, China.

**Methods:**

Genotyping of PPARD +294T/C polymorphism was conducted in 505 long-lived inhabitants (aged 90 and above, long-lived group, LG) and 468 healthy controls (aged 60–75, non-long-lived group, non-LG) recruited from Bama area.

**Results:**

No difference in allelic and genotypic frequencies was found between the two groups (*P* > 0.05). However, C-allele and C-genotype (TC and CC) were significantly more frequent in the females of non-LG than were LG after sex stratification. CC carriers exhibited higher LDL-C level in LG (*P* < 0.05) but lower TC, TG and LDL-C in non-LG (*P* < 0.05 for each) than TT carriers; C allele carriers (TC/CC) in LG exhibited higher TC, TG, and LDL-C levels as compared with the same genotype and the same lipid parameter in non-LG (*P* < 0.05 for each). LDL-C in LG was correlated with genotypes while TC, TG, and LDL-C in non-LG were correlated with genotypes (*P* < 0.05-0.001).

**Conclusion:**

Our results suggest that there were different impact patterns of PPARD +294T/C polymorphism on lipid profiles between long-lived cohort and average population in Bama area and this may be one of the genetic bases of its longevity.

## Introduction

The peroxisome proliferator-activated receptors (PPARs) belong to a superfamily of nuclear receptors. Three subtypes of PPARs, α, γ and δ, have been identified to date, with distinct roles in lipid and glucose metabolism. They are encoded by separate genes and characterized by distinct tissue-specific distribution patterns. PPARα and γ are primarily produced in the liver and adipocytes respectively and regulate fatty acid oxidation and adipogenesis accordingly, whereas PPARδ is ubiquitously expressed, with higher levels found in adipose tissue and skeletal muscle which might involve in the regulation of the β-oxidation of fatty acid [1-4]. In this context, disturbance in levels or activity of PPARs may elicit metabolic traits. In recent years, a number of efforts have thus been directed to the putative link between PPARs and age-related phenotypes such as metabolic syndrome, coronary heart disease (CHD) and Alzheimer’s disease (AD) [5,6], some of which have been well established both in animal and human models. Of these, PPARδ has been of burgeoning interest and proposed as a key player in the regulation of energy metabolism due to its ability to enhance fatty acid catabolism, energy uncoupling and insulin sensitivity in multiple tissues [7].

The PPARD gene that encodes human PPARδ is mapped to 6p21.2-p21.1 with 11 exons encompassing 35 Kbp [8]. Recent works have highlighted the potential roles for functional variants in PPARD gene in modulating its mRNA and protein levels which, in turn, affect genes regulated by PPARD [9-14]. Nine most common polymorphisms had been identified in PPARD: four in the intron, one in the 5′ untranslated region, and four in the 3′ untranslated region: c.-13598C > T, c.-13454G > T, c. + 294 T > C, c.285 + 700 T > C, c.285 + 793C > T, c.2022 + 12G > A, c.2022 + 351 T > C, c.2629 T > C, and c.2806C > G [9]. One of them, +294 T > C (rs2016520, also named -87 T > C or +15C > T), a T/C transition in nucleotide 15 of exon 4 located 87 base pairs before the start codon, is mostly studied in view of its critical role in lipid modulation [7,12]. The rare C allele versus the common T allele was shown to be associated with higher transcriptional activity and to influence binding of Sp-1 transcription factor [15]. Furthermore, subjects homogenous for C allele showed higher low density lipoprotein (LDL-C) level and a propensity towards a higher risk of CHD than T/T homozygotes [12,13,15-18], albeit conflicting results still exist in diverse populations [17-19]. Very recently, PPARδ was indicated to have anti-senescence activity in cultured human coronary artery endothelial cells [14]. Together, these results address that PPARD +294T/C polymorphism may play pivotal roles in the development of metabolic perturbations and subsequent aged-related pathologies, impacting consequently mortality and longevity in a given population. However, it is worth noting that the association studies between PPARD +294T/C polymorphism and serum lipid profiles were primarily undertaken in younger cohorts of European ancestry, data from population with exceptional longevity, particularly from China’s minorities, are evidently scarce. Bama long-lived individuals, a unique cohort reside along the midstream of Hongshuihe River in Guangxi Province, P. R. China, has emerged as an optimal cohort for human aging/longevity study in view of its low genetic background over the past decades [20]. Herein, we set out to test the hypothesis that the PPARD +294T/C polymorphism is associated with plasma lipid profile and longevity in Bama nonagenarians/centenarians of Zhuang ethnic origin.

## Materials and methods

### Study population

We studied 505 nonagenarians/centenarians (127 males and 378 females, age 93.29 ± 2.93 and range 90–104 years, referred to hereafter as the long-lived group or LG) who were recruited from Bama area (Bama, Fengshan, Donglan, and Du’an County) along the midstream of Hongshuihe River Basin, Guangxi Zhuang Autonomous Region, the People’s Republic of China. A total of 468 volunteers (212 males and 256 females, age 68.28 ± 4.66 and range 60–75 years) without a familial history of exceptional longevity (no past or current nonagenarian/centenarian in the first, second and third degree relatives) were randomly recruited as controls (non-long-lived group, non-LG) from the same geographic region. All subjects under investigation were unrelated and belong to Zhuang ethnic group, the China’s largest minority mainly living in Guangxi. All subjects were essentially healthy and had no evidence of any chronic illness, including hepatic, renal, or thyroid. The participants with a history of myocardial infarction, stroke, diabetes were also excluded. The current study was approved by the Ethics Committee of Guangxi Medical University. Informed consent was obtained from all subjects or their proxies after receiving full explanation of the study.

### Epidemiological survey

Socio-demographic information was obtained using a standardized questionnaire. Anthropometric variables including height, weight and waist were measured in all groups. Body mass index (BMI) was calculated as weight (kg)/height^2^ (m). Sitting blood pressure was measured 3 times, using a standard mercury sphygmomanometer with the subject resting for at least 5 minutes before measurement, and the average of the 3 measurements was used for the level of blood pressure. Systolic blood pressure was determined by the first Korotkoff sound; and diastolic, by the fifth Korotkoff sound. Hypertension was defined as systolic blood pressure > 140 mmHg and/or diastolic blood pressure > 90 mmHg. Normal weight, overweight, and obesity were defined as a BMI < 24, 24 to 28, and > 28 kg/m^2^, respectively [21].

### Biochemical measurements

A venous blood sample of 8 mL was drawn from each subject after an overnight fast, 4 mL of which was for serum separation and subsequent lipid determination while the remaining was transferred to an anticoagulant tube (4.80 g/L citric acid, 14.70 g/L glucose, and 13.20 g/L trisodium citrate) for DNA extraction. Total cholesterol (TC), triglycerides (TG), LDL-C and high density lipoprotein cholesterol (HDL-C) concentrations were measured by standard enzymatic methods using commercially available kits (Daiichi Pure Chemicals Co, Ltd., Tokyo, Japan) on a biochemical analyzer (Type 7170A; Hitachi Ltd, Tokyo, Japan) at the Clinical Science Experimental Center of the First Affiliated Hospital, Guangxi Medical University. The normal ranges of serum TC, TG, HDL-C, and LDL-C levels in the Center were 3.10-5.17, 0.56-1.70, 0.91-1.81, and 1.70-3.20 mmol/L, respectively. The individuals with TC > 5.17 mmol/L and/or TG > 1.70 mmol/L were defined as hyperlipidemic or dyslipidemia [22].

### Genotyping

**Figure 1:**
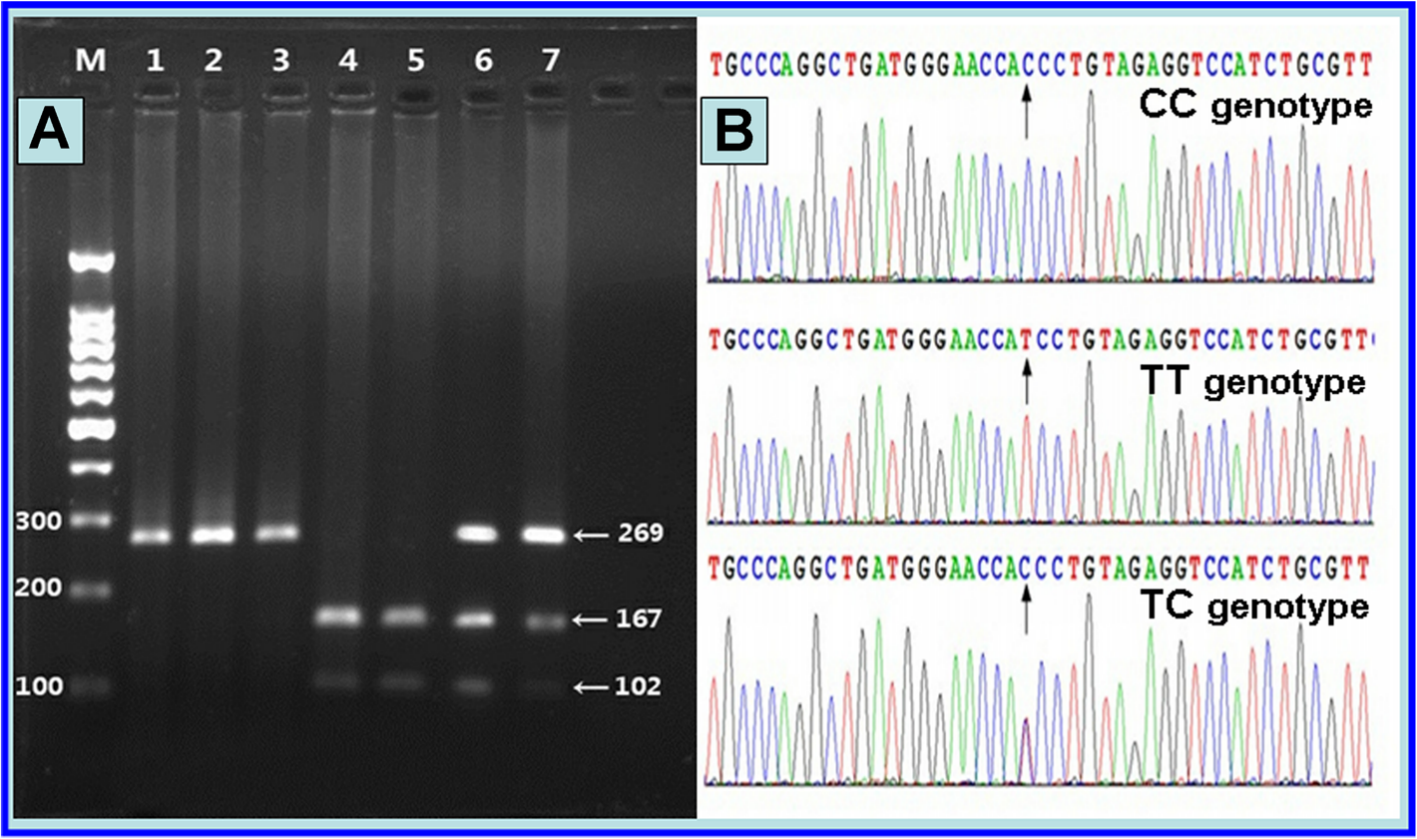
**Genotype results of PPARD +294T/C polymorphism. (A)** Lane M, 100 bp marker ladder; lanes 1, PCR product; lane 2, 3, TT genotype (269 bp); lane 4, 5, CC genotype (167- and 102-bp); and lanes 6, 7, TC genotype (269-, 167- and 102-bp). **(B)** Partial nucleotide sequences of PPARD +294T/C SNP. CC, TT, and TC genotypes.

Genomic DNA was extracted from white blood cells using standard methods [23]. Genotyping for the PPARD +294 T > C polymorphism was performed using polymerase chain reaction (PCR)-based restriction fragment length polymorphism (RFLP) as described by Wang et al. [24]. Briefly, a 269 bp fragment in the 5′-untranslated region of exon 4 of the PPARD gene was amplified by using primers 5′-CATGGTATAGCACTGCAGGAA-3′ (forward) and 5′-CTTCCTCCTGTGGCTGCTC-3′ (reverse) (Sangon Biotech, China). PCR was performed in a volume of 20 μL containing 200 ng of genomic DNA, 10 μL of Taq MasterMix (Beijing CoWin Bioscience, China), 6.25 μM (1.0 μL) of each primer, 7 μL ddH_2_O and 1 U of DNA polymerase [Takara Biotechnology, DaLian, China]. PCR conditions were: 95°C for an initial 5 min, followed by 35 cycles of 95°C 45 sec, 62°C 45 sec, and 72°C 45 sec, with a final 5 min extension at 72°C. The PCR products (10 μL) were digested with Bsl I (5 U) restriction endonuclease (New England Biolabs, Beijing, China) at 55°C overnight, and the fragments were separated on a 3% agarose gel containing ethidium-bromide and visualized with UV light. Band(s) at 269 bp only, at 269, 167, and 102 bp, and at 167, 102 bp indicate(s) TT, TC, and CC genotype, respectively (Figure 1A). To assess genotyping reliability, six samples (two for each genotype) were confirmed by direct sequencing in Sangon Biological Engineering Technology & Services Co., Ltd., China (Figure 1B). Laboratory technicians who performed genotyping were masked to clinical and biochemical data.

### Statistical analysis

Levels of the quantitative variables are presented as mean ± SD. Allelic and genotypic frequencies were calculated directly. Comparison of mean values of general characteristics between study groups and test for Hardy-Weinberg equilibrium were performed with the Pearson chi-square test. The statistical evaluation for the categorical variables was based on the calculation of the Student t-test. The association between the +294T/C polymorphism and lipid variables was tested by analysis of covariance (ANCOVA). Multiple logistic analyses with stepwise modeling were used to evaluate the association of serum lipid levels with genotypes (TT = 1, TC = 2, CC = 3) and several environment factors. All reported *P* values are from two-sided tests. A P-value < 0.05 was considered statistically significant. All analyses were performed using SPSS 13.0 (SPSS Inc, Chicago, IL).

## Results

### General characteristics and serum lipid levels

The basic demographic, clinical, and biochemical characteristics of participants of LG and non-LG are shown in Table 1. BMI was significantly lower in the LG as compared to non-LG group (*P* = 0.001). The levels of blood pressure, hypertension rate and unfavorable lipoprotein (TC, TG and LDL-C) were significantly higher (*P* < 0.01 for all) while the level of favorable lipoprotein (HDL-C) was slightly lower but did not reach statistical significance in LG than in controls. Despite the higher lipoprotein levels in LG, however, the prevalence of dyslipidemia was similar between LG and non-LG (*P* = 0.218).

**Table 1:** **Comparison of clinical characteristics and serum lipid profiles between LG and non-LG**

**Parameter**	**LG (n = 505)**	**Non-LG (n = 468)**	***t*** **(** ***χ*** ^***2***^ **)**	***P***
N (male/female)	127/378	212/256	43.445	0.000
Age (years)	93.29 ± 2.93	68.28 ± 4.66	41.967	0.000
Chest (cm)	79.38 ± 7.28	81.30 ± 7.21	−4.057	0.000
Waist (cm)	77.02 ± 9.52	75.73 ± 9.26	2.101	0.036
Hip (cm)	84.25 ± 6.62	87.06 ± 6.38	−6.588	0.927
BMI (kg/m^2^)	20.22 ± 3.43	20.94 ± 3.17	−3.401	0.001
SBP (mmHg)	166.23 ± 28.14	149.45 ± 25.31	9.792	0.000
DBP (mmHg)	89.21 ± 13.65	84.76 ± 12.28	5.342	0.000
TC (mM)	5.08 ± 1.01	4.91 ± 0.94	2.656	0.008
TG (mM)	0.97(0.49)	0.93(0.47)	1.878	0.006
HDL-C (mM)	1.58 ± 0.38	1.62 ± 0.37	−1.550	0.122
LDL-C (mM)	3.00 ± 0.86	2.82 ± 0.80	3.491	0.001
Dyslipidemia n(%)	240(47.52)	204(43.59)	1.516	0.218
Hypertension n(%)	425(84.16)	290(61.97)	61.396	0.000

LG, long-lived group; non-LG, non-long-lived group; BMI: body mass index; SBP, systolic blood pressure; DBP, diastolic blood pressure; TC, total cholesterol; TG, triglyceride; HDL-C, high-density lipoprotein cholesterol; LDL-C, low-density lipoprotein cholesterol. The values of triglyceride were presented as median (interquartile range) due to non-Gaussian distribution. The difference between the two groups was determined by the Wilcoxon-Mann–Whitney test.

### PPARD +294T/C polymorphism

The frequencies of the minor C-allele were 29.3% and 32.9% in LG and non-LG, respectively, and the genotypes were distributed according to the Hardy Weinberg Equilibrium in both groups. Overall, no significant difference was observed in the frequency distribution of genotypes and alleles between the two groups (both P > 0.05). When gender was taken into account, this distribution pattern remained similar in both sexes in intragroup comparison, but appeared to lose in females in intergroup comparison, with C-allele and TC/CC genotype tending to present more frequently in non-LG females than that in LG albeit the statistic powers were less robust (Table 2).

**Table 2:** **Comparison of genotypic and allelic frequency of the PPARD +294T/C polymorphism between LG and non-LG**

**Subgroup**	**n**	**Genotype n (%)**			**Allele n (%)**	
		**TT**	**TC**	**CC**	**T**	**C**
LG, all	505	261(51.70)	192(38.00)	52(10.30)	714(70.70)	296(29.30)
Non-LG, all	468	212(45.30)	204(43.60)	52(11.10)	628(67.09)	308(32.91)
*χ* ^*2*^				4.039		2.94
*P*				0.133		0.086
LG, male	127	58(45.70)	56(44.10)	13(10.20)	172(67.70)	82(32.30)
LG, female	378	203(53.70)	136(36.00)	39(10.30)	542(71.70)	214(28.30)
*χ* ^*2*^				2.835		1.451
*P*				0.242		0.228
Non-LG, male	212	100(47.20)	88(41.50)	24(11.30)	288(67.90)	136(32.10)
Non-LG, female	256	112(43.80)	116(45.30)	28(10.90)	340(66.40)	172(33.60)
*χ* ^*2*^				0.700		0.242
*P*				0.705		0.623
LG, male	127	58(45.70)	56(44.10)	13(10.20)	172(67.70)	82(32.30)
Non-LG, male	212	100(47.20)	88(41.50)	24(11.30)	288(67.90)	136(32.10)
*χ* ^*2*^				0.249		0.003
*P*				0.883		0.955
LG, female	378	203(53.70)	136(36.00)	39(10.30)	542(71.70)	214(28.30)
Non-LG, female	256	112(43.80)	116(45.30)	28(10.90)	340(66.40)	172(33.60)
*χ* ^*2*^				6.444		4.030
*P*				0.040		0.045

### Genotypes and serum lipid levels

In long-lived individuals, while levels of TC, TG, and HDL-C appeared similar across genotypes, there was a trend toward higher LDL-C in CC carriers relative to T carriers (TT/TC), but this tendency was no longer existed when analyses were stratified by sex. By contrary, in non-long-lived controls however, LDL-C tended to be lower in C allele carriers (TC/CC) than did TT carriers. Similar propensities were also observed on TC and TG levels in this group, even after sex was analyzed separately, especially in men. Intergroup comparison showed that TC, TG, and LDL-C levels of C allele carriers (TC and CC) but not TT carriers in LG, regardless of sex, were significantly higher than that of non-LG, implying that the minor C but not the wild type T allele may account in part for the difference of lipid profiles between LG and non-LG (Table 3).

**Table 3:** **Impact of PPARD +294T/C genotype on lipid levels**

**Subgroup**	**Genotype**	**n**	**TC (mmol/L)**	**TG (mmol/L)**	**HDL-C (mmol/L)**	**LDL-C (mmol/L)**
LG, all	TT	261	5.05 ± 0.99	0.96(0.49)	1.58 ± 0.39	2.97 ± 0.83
	TC	192	5.03 ± 1.01^b^	0.97(0.49)^b^	1.60 ± 0.36	2.97 ± 0.90^b^
	CC	52	5.40 ± 1.06^b^	1.10(0.48)^b^	1.54 ± 0.35	3.31 ± 0.83^ab^
	TC/CC	244	5.11 ± 1.03^b^	0.98(0.50)^b^	1.58 ± 0.36^b^	3.04 ± 0.89^b^
LG, male	TT	58	4.76 ± 1.04^c^	0.97(0.48)	1.52 ± 0.41	2.75 ± 0.95^c^
	TC	56	4.73 ± 1.07^c^	0.89(0.36)^c^	1.53 ± 0.40	2.77 ± 0.92
	CC	13	5.37 ± 1.00^d^	0.94(0.26)	1.64 ± 0.53	3.30 ± 0.61^d^
	TC/CC	69	4.85 ± 1.08^c^	0.92(0.33)^c^	1.55 ± 0.43	2.87 ± 0.89
LG, female	TT	203	5.13 ± 0.96	0.96(0.50)	1.60 ± 0.39	3.03 ± 0.78
	TC	136	5.15 ± 0.97^d^	1.02(0.64)^d^	1.62 ± 0.35	3.05 ± 0.88^d^
	CC	39	5.41 ± 1.09	1.15(0.56)	1.51 ± 0.27^d^	3.32 ± 0.90
	TC/CC	175	5.21 ± 1.00^d^	1.04(0.63)^d^	1.59 ± 0.33^d^	3.11 ± 0.89^d^
Non-LG, all	TT	212	5.04 ± 0.91	0.97(0.55)	1.61 ± 0.38	2.93 ± 0.80
	TC	204	4.77 ± 0.93	0.91(0.43)	1.63 ± 0.38	2.71 ± 0.79
	CC	52	4.92 ± 0.99^a^	0.88(0.53)^a^	1.64 ± 0.32	2.79 ± 0.82^a^
	TC/CC	256	4.80 ± 0.95^a^	0.89(0.44)^a^	1.63 ± 0.37	2.72 ± 0.79^a^
Non-LG, male	TT	100	4.96 ± 0.95	0.95(0.75)	1.57 ± 0.37	2.87 ± 0.85
	TC	88	4.65 ± 1.05	0.91(0.49)	1.58 ± 0.37	2.63 ± 0.87
	CC	24	4.59 ± 0.80^c^	0.88(0.37)^a^	1.52 ± 0.24^c^	2.66 ± 0.72
	TC/CC	112	4.64 ± 1.00^ac^	0.90(0.48)^a^	1.57 ± 0.35^c^	2.63 ± 0.84^a^
Non-LG, female	TT	112	5.11 ± 0.88	1.00(0.41)	1.65 ± 0.38	2.99 ± 0.76
	TC	116	4.86 ± 0.82	0.91(0.35)	1.66 ± 0.39	2.77 ± 0.72
	CC	28	5.21 ± 1.07	0.87(0.61)	1.74 ± 0.35	2.90 ± 0.90
	TC/CC	144	4.93 ± 0.88	0.89(0.43)^a^	1.68 ± 0.38	2.80 ± 0.75^a^

^a^ *P* < 0.05 in comparison with TT genotype of the same group; ^b^ *P* < 0.05 in comparison with the same genotype between LG and non-LG; ^c^ *P* < 0.05 intra-group comparison with the same genotype between sexes; ^d^ *P* < 0.05 intergroup comparison with the same genotype in males or females.

### Correlation between serum lipid parameters and genotypes

Multiple linear regression analyses showed that in the combined population, the four lipid parameters were mainly correlated positively with factors such as age, sex (female), BMI and diastolic blood pressure. When studied group was considered separately, LDL-C in LG was also noted to correlate positively with genotypes while TC, TG, and LDL-C in non-LG negatively with genotypes besides the above factors (Table 4). These findings are basically in line with results in Table 1 and Table 3.

**Table 4:** **Correlation between serum lipid parameters and PPARD +294T/C genotypes**

**Lipid**	**Relative factor**	**Unstandardized coefficient**	**Standard error**	**Standardized coefficient**	***t***	***P***
LG plus non-LG						
TC	DBP	0.012	0.002	0.158	4.905	0.000
	Gender	0.306	0.065	0.150	4.679	0.000
TG	BMI	0.009	0.002	0.176	5.337	0.000
	DBP	0.001	0.000	0.091	2.769	0.006
	Age	0.001	0.000	0.075	2.266	0.024
HDL-C	BMI	−0.012	0.004	−0.111	−3.375	0.001
	Gender	0.085	0.026	0.108	3.248	0.001
	Age	−0.003	0.001	−0.090	−2.688	0.007
LDL-C	DBP	0.007	0.002	0.115	3.468	0.001
	Gender	0.264	0.065	0.151	4.046	0.000
	Age	0.009	0.003	0.129	3.388	0.001
	Weight	0.010	0.004	0.114	2.661	0.008
LG						
TC	DBP	0.013	0.003	0.175	3.900	0.000
	Gender	0.344	0.104	0.149	3.318	0.001
TG	Weight	0.005	0.001	0.239	4.653	0.000
	Gender	0.078	0.019	0.213	4.150	0.000
HDL-C	Weight	−0.007	0.002	−0.147	−3.204	0.001
	DBP	0.002	0.001	0.091	1.981	0.048
LDL-C	DBP	0.008	0.003	0.130	2.878	0.004
	Gender	0.386	0.105	0.196	3.660	0.000
	Genotype	0.136	0.057	0.107	2.384	0.018
	Height	0.009	0.004	0.119	2.236	0.026
non-LG						
TC	Height	−0.016	0.005	−0.154	−3.244	0.001
	Waist	0.012	0.005	0.115	2.453	0.015
	Genotype	−0.148	0.064	−0.107	−2.309	0.021
	DBP	0.008	0.004	0.102	2.181	0.030
TG	BMI	0.022	0.004	0.383	5.601	0.000
	Hip	−0.006	0.002	−0.209	−3.057	0.002
	Genotype	−0.034	0.012	−0.130	−2.915	0.004
	DBP	0.002	0.001	0.111	2.444	0.015
HDL-C	Waist	−0.007	0.002	−0.168	−3.685	0.000
	Gender	0.108	0.035	0.143	3.118	0.002
	Age	−0.005	0.002	−0.121	−2.627	0.009
LDL-C	Waist	0.019	0.005	0.224	3.651	0.000
	SBP	0.004	0.001	0.122	2.657	0.008
	Genotype	−0.131	0.055	−0.109	−2.371	0.018
	Weight	−0.011	0.005	−0.127	−2.070	0.039

DBP: Diastolic blood pressure; SBP: Systolic blood pressure; BMI: Body mass index.

## Discussion

In the present study, the long-lived individuals exhibited significant higher TC, TG, and LDL-C levels than their younger counterparts. Higher mean age of the LG could not fully explain these differences. Our major concern is whether, and if so, to what extent the polymorphism of PPARD +294 involves in the modulation of lipid profile and longevity trait in these cohorts.

One of the important findings herein is the relatively higher frequency of the minor C allele of the PPARD +294 polymorphism, being 31.04% for the entire participants investigated, similar to that of healthy middle-aged Bai Ku Yao (22.50%) and Guangxi Han Chinese (27.57%) [17], two isolated subpopulations living in the branches of the upstream of Hongshuihe River, but noticeably higher than that of healthy Anhui Han Chinese (19.5%) [24], Korean (22.9%) [9], Swedish (15.6%) [15], Tunisian (18.9%) [18], Russian (12.1%) [25], German (19.2%) [19], and French-Canadians (20.1%) [11], demonstrating a population-specific allelic distribution pattern worldwide. The driving forces and the significance of this polymorphism in our studying population deserve further investigation.

The second finding in the current study is the marginal overrepresentation of minor C allele and genotypes (TC/CC) in the females of non-LG relative to that of LG (*P* = 0.040, 0.045, respectively, Table 2.) and the inverse relationship between PPARD +294C ratio and lipid levels in the serum in non-long-lived group. Hitherto, the associations between PPARD +294C and dyslipidemia, CHD, and other age-related pathologies have been replicated in several populations. For instance, elevated level of LDL-C was found in CC enotype carriers as compared to wild type TT homozygotes in Swedish healthy men [15], disease-free middle-aged Guangxi Han Chinese [17], and German mild obese and dyslipidemia patients [5]; the C-allele was significantly more frequent in Tunisian patients with cardiovascular artery disease than in controls [18]. Nevertheless, of interest to note is that subjects homozygous and heterozygous for PPARD +294C variant in non-LG had lower, instead of higher, levels of TC, TG and LDL-C. We have no exact explanation on these controversial results because, according to published literatures, the transition of nucleotide T to C at PPARD +294 may relate to either raised or no-change but not decreased level of unfavorable lipid.

In line with the preponderant view on the disadvantageous role that PPARD +294C may play in lipid modulation, we found a positive correlation between this polymorphism and lipid levels in long-lived group, which accounted for the third finding in our study. Thus, it seems reasonable that the females in LG present less C allele and CC genotype and this might confer them more opportunity to achieve longer lifespan than non-LG. However, we could not answer the question why the males in LG who presented similar C allele as general controls outlived their nineties.

Admittedly, lipid profiles and longevity are much more complex phenotypes which can not be interpreted with sole gene polymorphism. We speculate that the influence of PPARD + T294C on lipid metabolism, if any, may be limited, as do other lipid modulating genes such as cholesteryl ester transfer protein (CETP) [26] and microsomal triglyceride transfer protein (MTP) gene [20], each of which exerting small but pleiotropic effect on these complex traits, and this influence may concomitantly be affected by diet, lifestyle, and the interaction with other lipid related genes and environment via unraveled pathways.

To sum, our data show that inhabitants residing in Bama region of Guangxi, China display a higher minor allele frequency of the PPARD +294T/C polymorphism than other populations, with more C allele and C-containing genotypes representing in younger controls relative to long-lived individuals. Homozygous CC genotype is associated with increased LDL-C level in long-lived cohort whereas with decreased TC, TG and LDL-C levels in non-long-lived counterparts, demonstrating its age range-specific pattern on lipid profiles. The lower levels of unfavorable lipids in the average population in Bama area may relate to lower morbidity and mortality of age-associated diseases and this may in part pave the way to the longevity of the region. To the best of our knowledge, unlike others who mainly focused on younger healthy or patient populations, our research group is the first team to explore this polymorphism in long-lived and general individuals from a unique longevous region and report a reduced unfavorable lipids associated with PPARD +294C. However, longitudinal follow-up studies and gene functional studies are needed to further elucidate the precise effects of PPARD +294T/C polymorphism on serum lipids and longevity in Bama area.
